# Open-source environmental data as an alternative to snail surveys to assess schistosomiasis risk in areas approaching elimination

**DOI:** 10.1186/s12942-023-00331-w

**Published:** 2023-06-02

**Authors:** Elise N. Grover, William B. Allshouse, Andrea J. Lund, Yang Liu, Sara H. Paull, Katherine A. James, James L. Crooks, Elizabeth J. Carlton

**Affiliations:** 1 Department of Environmental and Occupational Health, Colorado School of Public Health, University of Colorado Anschutz Medical Campus, Aurora, USA; 2 Institute of Parasitic Diseases, Sichuan Center for Disease Control and Prevention, Chengdu, China; 3 Department of Epidemiology, Colorado School of Public Health, University of Colorado Anschutz Medical Campus, Aurora, USA; 4 Division of Biostatistics and Bioinformatics, National Jewish Health, Denver, USA

**Keywords:** Schistosomiasis, Geographic information systems, Remote sensing technology, Machine learning, Prevention and control, China, Infectious disease surveillance, Snails, *Oncomelaniahupensis*

## Abstract

**Background:**

Although the presence of intermediate snails is a necessary condition for local schistosomiasis transmission to occur, using them as surveillance targets in areas approaching elimination is challenging because the patchy and dynamic quality of snail host habitats makes collecting and testing snails labor-intensive. Meanwhile, geospatial analyses that rely on remotely sensed data are becoming popular tools for identifying environmental conditions that contribute to pathogen emergence and persistence.

**Methods:**

In this study, we assessed whether open-source environmental data can be used to predict the presence of human *Schistosoma japonicum* infections among households with a similar or improved degree of accuracy compared to prediction models developed using data from comprehensive snail surveys. To do this, we used infection data collected from rural communities in Southwestern China in 2016 to develop and compare the predictive performance of two Random Forest machine learning models: one built using snail survey data, and one using open-source environmental data.

**Results:**

The environmental data models outperformed the snail data models in predicting household *S. japonicum* infection with an estimated accuracy and Cohen’s kappa value of 0.89 and 0.49, respectively, in the environmental model, compared to an accuracy and kappa of 0.86 and 0.37 for the snail model. The Normalized Difference in Water Index (an indicator of surface water presence) within half to one kilometer of the home and the distance from the home to the nearest road were among the top performing predictors in our final model. Homes were more likely to have infected residents if they were further from roads, or nearer to waterways.

**Conclusion:**

Our results suggest that in low-transmission environments, leveraging open-source environmental data can yield more accurate identification of pockets of human infection than using snail surveys. Furthermore, the variable importance measures from our models point to aspects of the local environment that may indicate increased risk of schistosomiasis. For example, households were more likely to have infected residents if they were further from roads or were surrounded by more surface water, highlighting areas to target in future surveillance and control efforts.

**Supplementary Information:**

The online version contains supplementary material available at 10.1186/s12942-023-00331-w.

## Background

The water-borne disease, schistosomiasis, has been targeted by the World Health Organization for elimination as a public health problem by the year 2030 in a total of 78 endemic countries, where decades-long control programs have led to major reductions in infections and morbidity in many of them [1]. However, as transmission becomes more sporadic as a result of successful disease control programs, surveillance strategies also need to be recalibrated to allow efficient identification of pockets of on-going infection at fine spatial scales so that these areas can be targeted for treatment and transmission-blocking interventions.

Surveillance of schistosomiasis is difficult due to the gradual onset of disease, and non-specific, intermittent symptoms such as abdominal pain, diarrhea and rectal bleeding [2]. When left untreated, infection can lead to a range of serious conditions including stunted childhood development and cognitive impairment, anemia, pulmonary hypertension, fibrosis of vital organs, and in the most serious cases, death [2]. The slow and non-specific disease onset means infected individuals rarely seek care upon infection, and thus passive clinic and/or hospital-based surveillance, widely used for other infectious diseases, are not reliable ways to monitor infections. Notably, some naïve individuals develop acute morbidity upon infection, due to an inflammatory reaction to the migrating schistosome [3]. Acute schistosomiasis, or Katayama fever, can signal emerging infections, but reliance on acute case reporting alone will lead to misclassification of many areas with ongoing transmission [4].

Malacological surveys for the presence of the intermediate snail host and schistosomiasis infections in snails are a common schistosomiasis surveillance tool used in endemic countries worldwide [5–12]. Schistosomiasis transmission is highly influenced by environmental conditions, as the presence of an infected intermediate snail host is a necessary precondition for transmission to humans and other vertebrate hosts [13]. The significance of the ambient environment in the schistosomiasis transmission cycle is heightened by the fact that the lifecycle involves two key timepoints when the developing parasite must survive in open water, moving from a mammalian host’s feces to an intermediate snail host during the miracidia stage, and later swimming from an intermediate snail to a new mammalian host during the cercarial stage [2, 14, 15]. Thus, a combination of environmental conditions—including soil and vegetative health, the presence of fresh water, temperature, season and elevation—can impact the likelihood of snail habitation, the survival of the parasite, and the overall transmission potential of a given location [15–18].

Despite the key role that snails play in the transmission of schistosomiasis, using them as surveillance targets is challenging due to the patchy and dynamic quality of snail habitats and the sparsity of snail infections. Identifying, collecting and testing snails for *Schistosoma* infections is time-consuming and labor-intensive requiring surveying kilometers of transects, collecting thousands of snails and repeating surveys to account for seasonal fluctuations in snail populations [19, 20]. Although infected snails are a necessary condition for mammalian schistosome infection to occur, they are often poor predictors of human infection risk [4, 19–21]. For example, assessments conducted in endemic provinces of China between 2016 and 2017 did not find any *Schistosoma japonicum* infected snails from several million that were systematically identified, collected and tested during comprehensive snail survey efforts, despite having identified low to moderate levels of infection in humans and other mammalian hosts [22–24]. Similarly, while the presence of intermediate snail hosts has been broadly correlated with human and livestock infection in some instances [25, 26], the transient, impermanent nature of snail habitats can also make them an inconsistent predictor of human infection risk and an unreliable target for schistosomiasis surveillance [20].

As a result, assessments of schistosomiasis transmission environments have increasingly relied on measures of environmental characteristics, often using remote sensing in combination with geospatial analyses. There is a considerable body of literature demonstrating the use of climate and environmental variables (e.g., humidity, precipitation, temperature, elevation, vegetation, distance to the nearest waterbody, etc.) to estimate environmental suitability for snail habitation (e.g., 16, 18, 26–29), which can theoretically be used to highlight potential schistosomiasis hotspots. However, few studies have demonstrated the use of environmental characteristics to directly predict human schistosomiasis risk [17, 20, 30]. A recent study in Senegal found that measures of vegetation and water contact area were better predictors of *S. haematobium* reinfection in children in a highly endemic region than measures collected during on-the-ground snail surveys [20]. Similarly, studies of *S. japonicum* infection in China have found measures of vegetation and proximity to rivers were predictive of human infection clusters [17, 30]. In all three studies, the models were designed to identify infections at the village scale. We see the need for higher resolution environmental proxies of human schistosomiasis in low transmission settings, such that pockets of potential infection could be identified at the household or neighborhood level.

As regions approach schistosomiasis elimination goals, the perceived payoff of comprehensive infection and snail surveys will decrease, making it likely that resources will be diverted to other priorities in the coming decades. In order to avoid a resurgence in schistosomiasis, it is crucial that cost-effective, low labor surveillance techniques are developed that can be used to pinpoint, at fine geographic scales, areas of high infection risk in areas approaching elimination. Precision risk mapping can enable targeting of resources to high-risk areas for testing, treatment or transmission-blocking interventions. The proliferation of high-resolution, open-source geospatial data products offer an opportunity to develop new methods for mapping schistosomiasis risk in areas where control programs have reduced but not fully eliminated schistosomiasis.

The primary aim of this analysis was to determine whether open-source environmental data that is freely available and less time- and labor-intensive to collect than snail survey data can directly predict household schistosomiasis infection distribution, with a similar or improved degree of accuracy as data obtained during snail surveys. To do this, we developed and compared two models for predicting household *S. japonicum* infection among rural farming communities in Sichuan Province, China. In our first model, we used geocoded snail survey data to build a set of predictors and determine how well the proximity and density of snail habitat relative to the location of the home predict household *S. japonicum* infection status. In the second model, we drew on freely available, open-source environmental data to create a set of measures characterizing local environmental conditions in the area surrounding the home in the months prior to infection surveys. By comparing the ability of these two models to predict fine-scale geographic patterns of human *S. japonicum* infection, our study provides valuable information on the utility of each of these surveillance techniques for identifying potential high- and lowrisk households in communities where low levels of persistent *S. japonicum* infection are obstructing elimination goals. As a secondary analysis, we evaluated the relative importance and the direction of association between our predictors and household *S. japonicum* infection to shed light on those characteristics of the local environment that can be leveraged for prediction modeling in the study area and targeted in future prevention and control efforts.

## Methods

### Setting and village selection

**Fig. 1:**
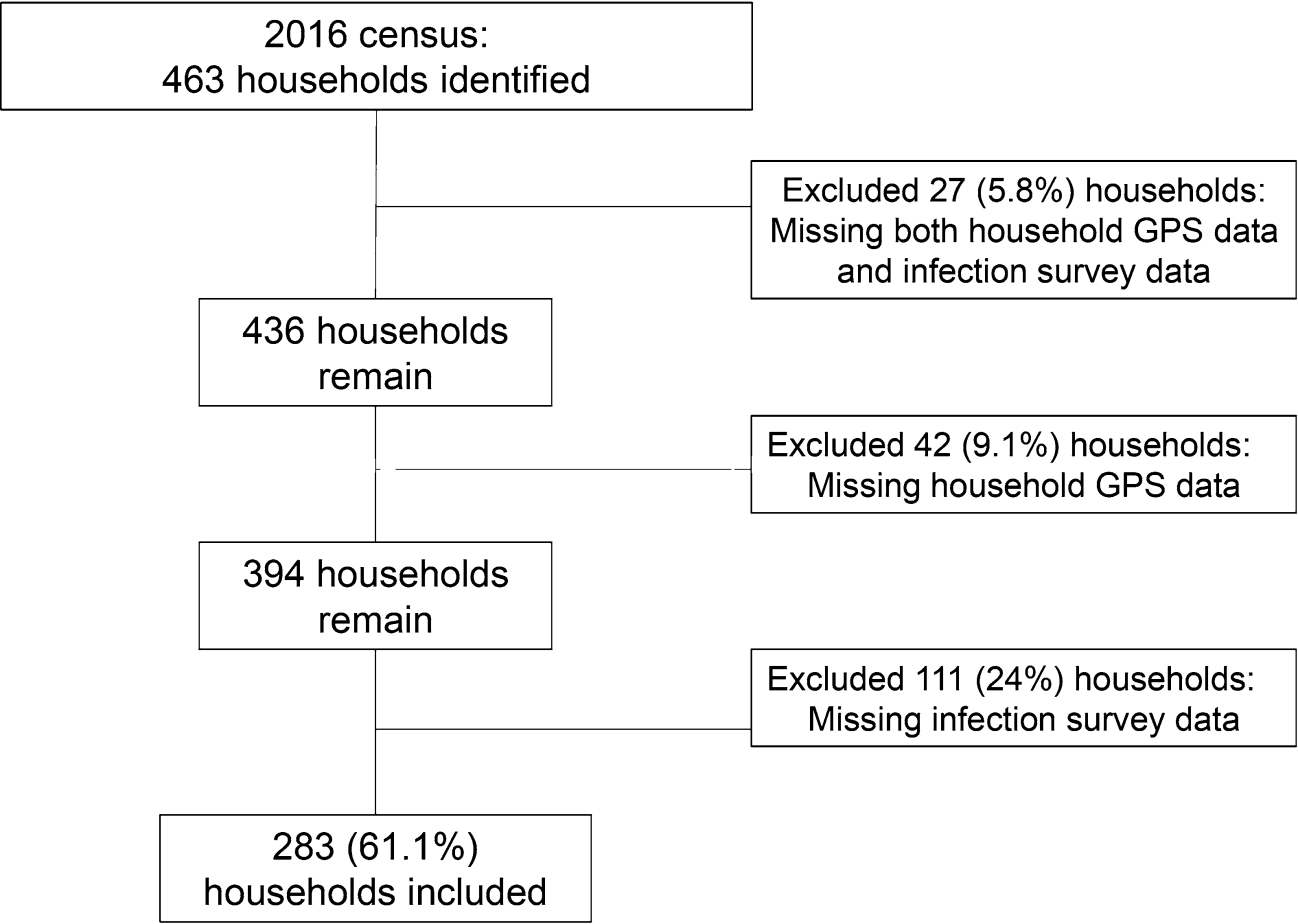
Depiction of household inclusion and exclusion

This study was conducted in 2016 in two counties located in the hilly regions of Sichuan Province, China. County surveillance records were used to select ten villages at high risk of reemergent or ongoing *S. japonicum* transmission. We conducted a census in each village, attempted to geocode the location of all households using handheld Global Positioning System (GPS) devices, and surveyed each household for *S. japonicum* infection, as described below. The number of households in the selected villages ranged from 19 to 75, with between 50 to 250 residents residing in each village at the time of data collection. We restrict this analysis to households for which: (a) GPS coordinates were successfully recorded, and (b) at least one household resident was tested for *S. japonicum* infection during the 2016 infection surveys. Of the 463 households identified during the census, a total of 283 households (61.1%) had both GPS and infection survey data and are therefore included in this analysis. See Fig. 1 for details on household exclusion and inclusion.

### Data collection and sources

Human infection data were collected in July 2016 as a part of ongoing research efforts in the region assessing persistent schistosomiasis hotspots. All village residents over the age of five were invited to participate in the study. Each participating individual was asked to provide three stool samples on consecutive days. All samples were labelled with the date of collection and participant ID numbers and stored in a cooler or cool room (ideally < 10 °C) until they could be transported to the central laboratory for processing. Samples were examined using the miracidium hatching test, following standard protocols [31]. In brief, for each sample, 30 g of stool was suspended in water (pH range of 6.8–7.2), strained to remove large particles (80 head nylon mesh), strained again to retain the remaining solids (280 head nylon mesh), and then suspended in water at room temperature (28–30 °C). At 2-, 4- and 8- hours after suspension, the samples were examined for the presence of miracidia for at least 2-min each time. An individual was considered positive if any of the three hatch tests were positive.

The habitats of *Oncomelania hupensis* snails (the intermediate host that transmits *S. japonicum* to humans and other vertebrate hosts) were determined during a national survey on *O. hupensis* conducted in 2016. Snail habitats were first identified by trained professionals from county anti-schistosomiasis control stations using historical records dating back to the 1950s. Historical habitats were digitized by global positioning and geographic information systems (GIS). Surveys were then conducted in the field via transect walks between the months of April and October 2016 using standard systematic sampling methods [32]. Briefly, each historic, existing or suspected snail environment was divided into sampling frames set every 5–10 m, with parallel lines extending from each to form a set of sampling frames of between 25–100 m^2^ covering each site. The majority of existing or suspected sites were characterized by shallow, stagnant or moving water (e.g. a stream, pond, rice paddy or irrigation ditch), as these conditions are the preferred habitat of amphibious freshwater *O. hupensis* snails [33]. For each site, ~ 20% of the sampling frames were randomly selected to be investigated on foot for the presence of snails. The digitized maps were updated using handheld GPS devices to document present and absent habitat locations, shapes, and whether and how historic habitats had been destroyed or changed (e.g., land use change via urbanization).

Data on waterbodies, waterways and roads in Sichuan Province, China were obtained on November 11th, 2021 from Geofabrik, a company that specializes in OpenStreetMap (OSM) data [34]. The OSM project draws on local communities of mappers to build a knowledgeable database detailing roads, waterways, transportation and other built and natural environment features [35]. OSM Contributors use aerial imagery, handheld GPS devices, and field maps, both to generate the data and to verify the accuracy of the open data on a regular basis [35]. OSM data on waterways and waterbodies include permanent water features such as large rivers, streams, canals, lakes and reservoirs, while roads data ranges from national freeways and motorways down to gravel tracks and paths. Although OSM coverage can be low in China for private roads, roads for non-motorized vehicles and residential roads, OSM coverage is high for features like highways and main roads (> 80%) [36]. As such, data included from OSM in this analysis primarily represents main roads and major environmental features. Details on the OSM data used in this analysis can be found at: https://download.geofabrik.de/osm-data-in-gis-formats-free.pdf [37].

Elevation Data was obtained from the Earth Observation Research Center Japan Aerospace Exploration Agency’s (JAXA EORC) Advanced Land Observing Satellite (ALOS) global digital surface model, which has a horizontal resolution of approximately 30 m [38]. To calculate indices of vegetation and waterbody coverage, the U.S. Geological Survey’s (USGS) Earth Resources Observation and Science (EROS) Center’s image library from the Landsat Satellite 8 – Collection 1 was accessed from the USGS Earth Explorer website (https://earthexplorer.usgs.gov/) to obtain data on surface reflectance bands 2–5, as well as the QA band [39]. The Landsat-8 satellite repeats its orbital pattern every 16-days [40], resulting in a total of 12 available observations across 2016 that occurred prior to our July infection surveys, which were downloaded for use in this study. The National Aeronautics and Space Administration’s (NASA) pre-processed Moderate Resolution Imaging Spectroradiometer (MODIS) Terra satellite imagery database was also accessed to obtain 250-m resolution data on vegetation at 16-day intervals at all available timepoints in 2016 prior to the infection surveys [41].

## Variable definitions and generation

### Outcome variable

*S. japonicum* infection survey results from the ten study villages were aggregated to the household level and spatially joined to the geographic location of the home. To avoid issues with multicollinearity resulting from residents of the same household having the same values for all environmental predictors used in this analysis, the outcome was a binary measure of household infection status indicating whether one or more household member tested *S. japonicum* positive.

### Predictor variables: snail survey dataset

Using the 2016 snail survey data, predictors were generated to reflect how a household’s position in relation to snail habitats could influence household-level *S. japonicum* infection risk (Table 1). The geocoded snail habitat data was divided into two categories: present snail habitat sites, and absent snail habitat sites. Present snail habitat sites were those sites where one or more snails were identified during the survey period, while absent snail habitat sites were those where snails were not found during the 2016 survey. The data were further grouped into “ditches” (i.e., line features deemed suitable for snail habitation) and “fields” (i.e., polygon features deemed suitable for snail habitation), resulting in four snail habitat categories: present ditches, present fields, absent ditches, and absent fields. Using ArcGIS Pro software [42], three different buffer sizes (0.25, 0.5 and 1.0 km (km) radius length) were generated and applied to each household location. Buffer radius lengths were defined such that the largest buffer (1 km) generally spanned the entire village area for a centrally located household, whereas the smallest buffer (0.25 km) spanned the immediate surroundings of a household. We generated variables estimating the density of ditches and fields surrounding the home by calculating the total length (km) of present and absent ditches and the total area of present and absent fields (km^2^) within each of buffer. The geodesic distances (m) between each household point and the nearest present ditch, absent ditch, present field, and absent field were also calculated and used as predictors.

**Table 1:** List of predictors generated for each model

Snail survey data model	Environmental predictors model
**Presence sites (one or more snails found)**	**Open data**
* Ditches where snails were present*	*Built and natural environment*
Distance from home to the nearest identified present ditch (km)	Distance to nearest waterway (km)
Total length of present ditches within 0.25 km radius of the home (km)	Distance to nearest waterbody (km)
Total length of present ditches within 0.5 km radius of the home (km)	Distance to nearest road (km)
Total length of present ditches within 1 km radius of the home (km)	Elevation (m)
*Fields where snails were present*	
Distance from home to the nearest identified present field (km)	**Remotely sensed data**
Total area of present fields within 0.25 km radius of the home (km^2^)	*Normalized Difference Water Index (NDWI)*
Total area of present fields within 0.5 km radius of the home (km^2^)	Average NDWI within 0.25 km radius of the home
Total area of present fields within 1 km radius of the home (km^2^)	Average NDWI within 0.5 km radius of the home
	Average NDWI within 1 km radius of the home
**Absence sites (no snails found)**	*Normalized Difference Vegetation Index (NDVI)*
* Ditches where snails were absent*	Average NDVI within 0.25 km radius of the home
Distance from home to the nearest identified absent ditch (km)	Average NDVI within 0.5 km radius of the home
Total length of absent ditches within 0.25 km radius of the home (km)	Average NDVI within 1 km radius of the home
Total length of absent ditches within 0.5 km radius of the home (km)	*Enhanced Vegetation Index (EVI)*
Total length of absent ditches within 1 km radius of the home (km)	Average EVI within 0.25 km radius of the home
*Fields where snails were absent*	Average EVI within 0.5 km radius of the home
Distance from home to the nearest identified absent field (km)	Average EVI within 1 km radius of the home
Total area of absent fields within 0.25 km radius of the home (km^2^)	
Total area of absent fields within 0.5 km radius of the home (km^2^)	**Other predictors included in the models**
Total area of absent fields within 1 km radius of the home (km^2^)	Number of people tested in the household (N)
**Other predictors included in the models**	
Number of people tested in the household (N)	

For the snail survey data models, present sites are those where at least one snail was found, while absent sites are those where no snails were found during the 2016 snail surveys. For the environmental data models, NDVI and NDWI were calculated using Landsat-8, Collection 1 satellite data collected on January 23rd, February 8th, and April 28th (dates where there was < 30% cloud cover). Pre-processed EVI data from NASA’s Moderate Resolution Imaging Spectroradiometer (MODIS) Terra satellite was averaged across a total of 12 observations occurring at 16-day intervals between January 1st and July 10th, 2016

*NDWI* Normalized Difference Water Index, *NDVI* Normalized Difference Vegetation Index, *EVI* Enhanced Vegetation Index

### Predictor variables: open-source environmental dataset

Open-source environmental and remotely sensed data were compiled to create a geospatial dataset containing a range of hypothesized environmental (built and natural) predictors of household *S. japonicum* infection (Table 1). Potential environmental predictors were selected if they were (1) previously identified or hypothesized in the literature to serve as predictors of schistosomiasis infection or snail habitat sites; and (2) made publicly available at a 250-m resolution or finer for the entire study area. The elevation (m) of the home and the geodesic distance (km) to the nearest road, waterway and waterbody was calculated for each household. We generated the average Normalized Difference Water Index (NDWI) [43] and average Normalized Difference Vegetation Index (NDVI) [44] as estimates of water content and vegetation health in our study area, respectively, using 30-m resolution Landsat-8 satellite images with less than 30% cloud cover collected between January and July 2016. Whereas the NDWI identifies water features and distinguishes them from soil and vegetation surfaces [43], the NDVI is chlorophyll-sensitive and provides a measure of crop and vegetation health [45]. We calculated an additional measure of vegetation frequently used in high biomass regions due to its sensitivity to variations in canopy health [45], the average Enhanced Vegetation Index (EVI) [46]. This was estimated for the period between January and July 2016 using NASA’s MODIS data library providing pre-processed 250-m resolution EVI data [41]. As was done for the snail data, three different buffers sizes (0.25, 0.5 and 1.0 km) were generated around each household point, and the average NDWI, NDVI and EVI were calculated for each. Not only did this make our measures of NDVI, NDWI and EVI representative of the average conditions surrounding the home, but this also helped to facilitate the comparison between our 30-m resolution measures of NDWI and NDVI and the 250-m resolution measure of EVI. A detailed description of variable rationale and definitions, and the process and ArcGIS Pro tools used to form each of them are provided in Additional File 1.

## Analysis

### Primary analysis

A Random Forests (RF) machine learning approach was used to construct and compare predictive models, one using snail survey data and one using open-source environmental data as predictors. After generating our datasets in ArcGIS Pro, the R-ArcGIS Bridge from the ‘arcgisbinding’ R package was used to facilitate an easy transfer of data between ArcGIS and RStudio for the RF analysis [47]. Each dataset was split 75/25 for training and validation, respectively. For each training dataset, we oversampled the minority class to correct for class imbalance in our outcome variable (13.8% of households were *S. japonicum* positive). In total, three different balanced training datasets were generated for the snail data, and three for the environmental data, yielding a total of six balanced datasets that were used for RF model training. This approach allowed us to assess the stability of model performance metrics and variable importance rankings in light of our oversampling approach. The ‘caret’ package in R was used to perform a tenfold cross validation process to tune each model, helping to determine the optimal maximum node size to use and the number of variables to try at each branch. For each RF model, we specified 5000 trees per forest, as a high number of trees is recommended to help stabilize variable importance rankings [48].

The reserved validation data was used to test each model and calculate performance statistics (accuracy, Cohen’s kappa statistic, receiver operator curve (ROC) area under the curve (AUC), sensitivity, specificity, positive predictive value (PPV) and the negative predictive value (NPV)). To compare performance between models, the best model was defined as the one with the highest kappa value, followed by accuracy and ROC AUC, respectively. Because our reserved validation datasets had a high degree of class imbalance, the kappa statistic was selected as our main metric for indicating model performance, as it was developed to help correct for bias related to over-rewarding the prediction of the majority class [49]. Model accuracy was also compared to the No Information Rate (NIR), which indicates what the accuracy would be expected to be if the majority class were predicted every time (NIR = 0.859). A high NIR value results when there is a high degree of class imbalance for the outcome of interest, as was the case in this study. Finally, in the event of a tie in the kappa and accuracy of two models, the ROC AUC was used to select a final, top performing model.

### Secondary analysis

To determine which predictors were the most influential in predicting household *S. japonicum* infection, a secondary analysis was performed using the mean decrease in accuracy (MDA) values of predictors to visualize relative variable importance within each model. For each of the three environmental data models and three snail data models, the top ten predictors indicated by the model’s MDA plots were given a score of 10 to 1 (10 being the score of the top predictor). Variable scores were then summed across the three models to create a three-model summary score of 0 to 30, 30 being the highest score possible, while a score of 0 indicates that the variable was never ranked among the top ten predictors. Simple logistic regression models and lowess plots were examined to determine the direction and shape of association between household *S. japonicum* infection status and each predictor.

As a demonstration of the application of our approach, a map of the predicted probability of *S. japonicum* infection was generated using the top performing RF model indicated by the primary analysis to highlight high-risk locations and features in our study area in 2016. All analyses were conducted in ArcGIS Pro 2.8.3 and RStudio Version 4.1.2 [42, 47].

## Results

**Fig. 2:**
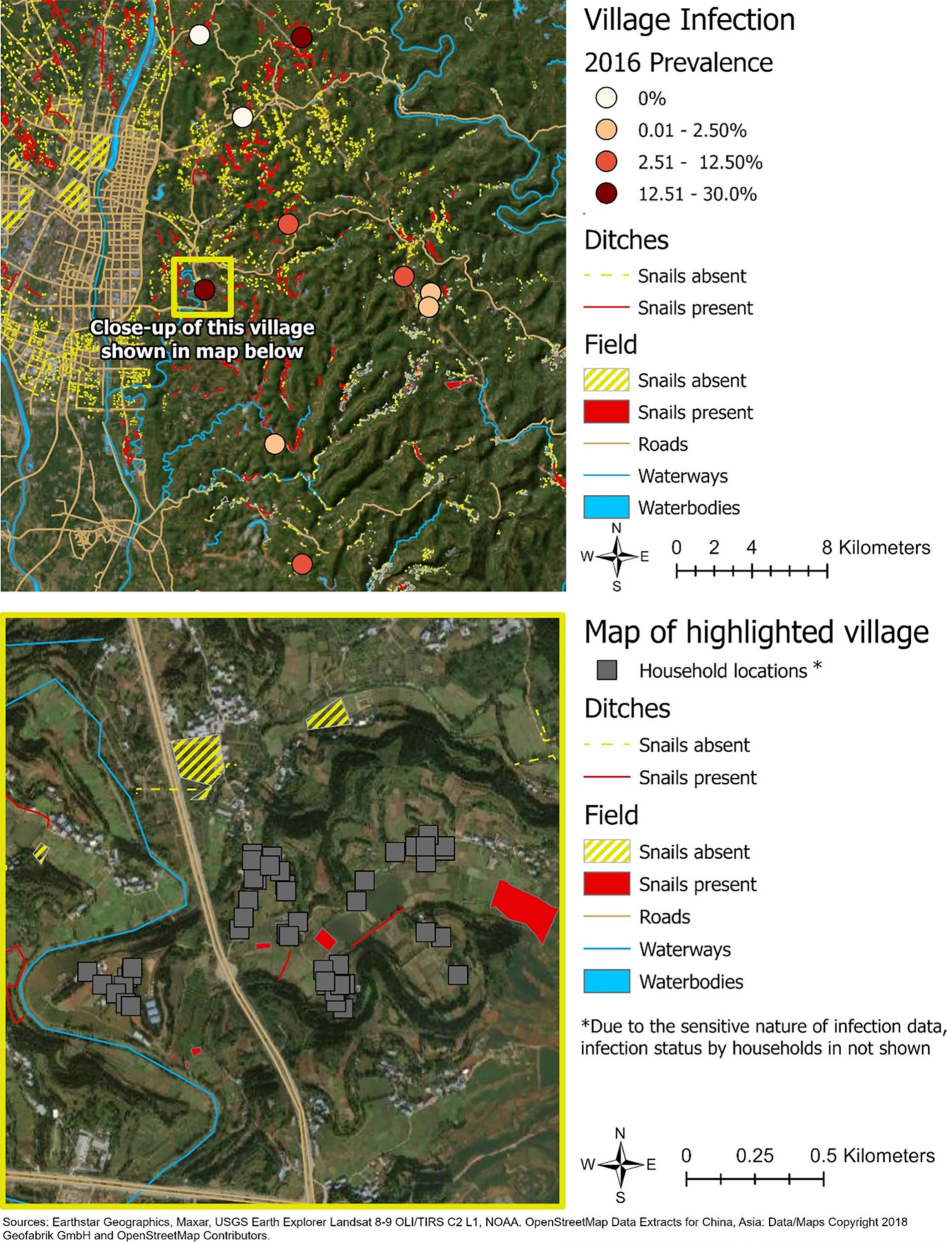
Maps of the study villages

Village-level *S. japonicum* infection prevalence (n = 10) ranged from 0% to 27.1%, while the number of infections per household ranged from 0 to 3, with a mean of 0.16 (Standard Deviation (SD) = 0.44) infections per household across the 283 households. A total of 4,896 historical or current snail habitat sites were identified in the study area, of which 1,092 (22.3%) were found to contain one or more snails. None of the snails identified during the snail surveys were found to be infected with *S. japonicum*. In total, 69.7% of sites were categorized as ditches. The total length of ditches within 1 km of the home ranged from 0 to 7.31 km long, with an average length of 1.74 km (SD = 1.70) for ditches with snails present, and 2.22 km (SD = 1.35) for ditches where snails were absent. The remaining 30.3% of the surveyed sites were categorized as fields. Within 1 km of the home, the total area of fields (present or absent) ranged from 0 to 0.19 km^2^. The average area of fields with snails present was 0.04 km^2^ (SD = 0.06) and 0.06 km^2^ (SD = 0.06) for fields where snails were absent. Figure 2 illustrates the geographic distribution of infections in relation to the snail habitat sites for our study area.

On average, the homes in our study villages were located closer to a road (mean distance: 0.36 km) than to a waterbody (2.11 km) or waterway (3.02 km). The mean elevation of households in the study villages was 573 m. Surface water in the area surrounding the home was generally low. NDWI values can range from -1 to 1, with a value of < 0 indicating a surface with little to no water content, though a threshold of > 0.3 has been proposed as a reasonable value to use for identifying waterbodies [50]. In our study, the mean NDWI within 1 km of the home was − 0.19 (SD = 0.01). Similarly, the NDVI and EVI range from −1 to 1, with lower values indicating more barren landscapes. Values lower than 0.1 for NDVI represent low vegetation areas (e.g. rocks, sand or snow), while values greater than 0.6 corresponds with temperate and tropical forests [51]. For the EVI, values between 0.2 and 0.8 are generally used to indicate healthy vegetation [52]. The average NDVI and EVI within 1 km of the home was 0.18 (SD = 0.02) and 0.40 (SD = 0.02), respectively. Table 2 provides summary statistics for the household predictors included in this analysis.

**Table 2:** Summary of household variables

household variables	N	%		
**Shared model characteristics**				
Number of infections per household				
0	244	86.22		
1	33	11.66		
2 +	6	2.12		
Number of people tested per household				
1	83	29.33		
2	146	51.59		
3	42	14.84		
4 +	12	4.24		
**Snail models**	Mean	SD	Min.	Max.
*Ditches*				
Distance from home to nearest present ditch (km)	0.57	0.89	< 0.01	3.26
Length of present ditches within 0.25 km of the home (km)	0.24	0.28	0.00	0.97
Length of present ditches within 0.5 km of the home (km)	0.65	0.61	0.00	2.69
Length of present ditches within 1 km of the home (km)	1.74	1.70	0.00	7.31
Distance from home to nearest absent ditch (km)	0.26	0.23	< 0.01	1.05
Length of absent ditches within 0.25 km of the home (km)	0.23	0.28	0.00	0.93
Length of absent ditches within 0.5 km of the home (km)	0.77	0.65	0.00	2.11
Length of absent ditches within 1 km of the home (km)	2.22	1.35	0.00	6.80
* Fields*				
Distance from home to nearest present field (km)	0.58	0.51	< 0.01	1.55
Area of present fields within 0.25 km of the home (km^2^)	0.01	0.01	0.00	0.05
Area of present fields within 0.5 km of the home (km^2^)	0.02	0.04	0.00	0.13
Area of present fields within 1 km of the home (km^2^)	0.04	0.06	0.00	0.16
Distance from home to nearest absent field (km)	0.36	0.54	< 0.01	2.20
Area of absent fields within 0.25 km of the home (km^2^)	0.01	0.01	0.00	0.19
Area of absent fields within 0.5 km of the home (km^2^)	0.02	0.02	0.00	0.09
Area of absent fields within 1 km of the home (km^2^)	0.06	0.06	0.00	0.04
**Environmental data models**	Mean	SD	Min.	Max.
*Built and natural environment*				
Distance from home to the nearest waterway (km)	3.02	1.82	0.06	5.44
Distance from home to the nearest waterbody (km)	2.11	0.95	0.37	3.91
Distance from home to the nearest road (km)	0.36	0.26	< 0.01	1.27
Elevation of the home (m)	573.45	54.81	495.0	685.0
*Remotely sensed data*				
Mean NDWI within 0.25 km of the home	− 0.19	0.02	− 0.23	− 0.15
Mean NDWI within 0.5 km of the home	− 0.19	0.02	− 0.21	− 0.16
Mean NDWI within 1 km of the home	− 0.19	0.01	− 0.20	− 0.17
Mean NDVI within 0.25 km of the home	0.18	0.02	0.14	0.23
Mean NDVI within 0.5 km of the home	0.18	0.02	0.14	0.22
Mean NDVI within 1 km of the home	0.18	0.02	0.15	0.21
Mean EVI within 0.25 km of the home	0.40	0.03	0.36	0.48
Mean EVI within 0.5 km of the home	0.40	0.02	0.37	0.47
Mean EVI within 1 km of the home	0.40	0.02	0.38	0.46

*NDWI* Normalized difference Water Index, *NDVI* Normalized difference Vegetation Index, *EVI* Enhanced Vegetation Index

## Primary analysis

### RF model performance

**Fig. 3:**
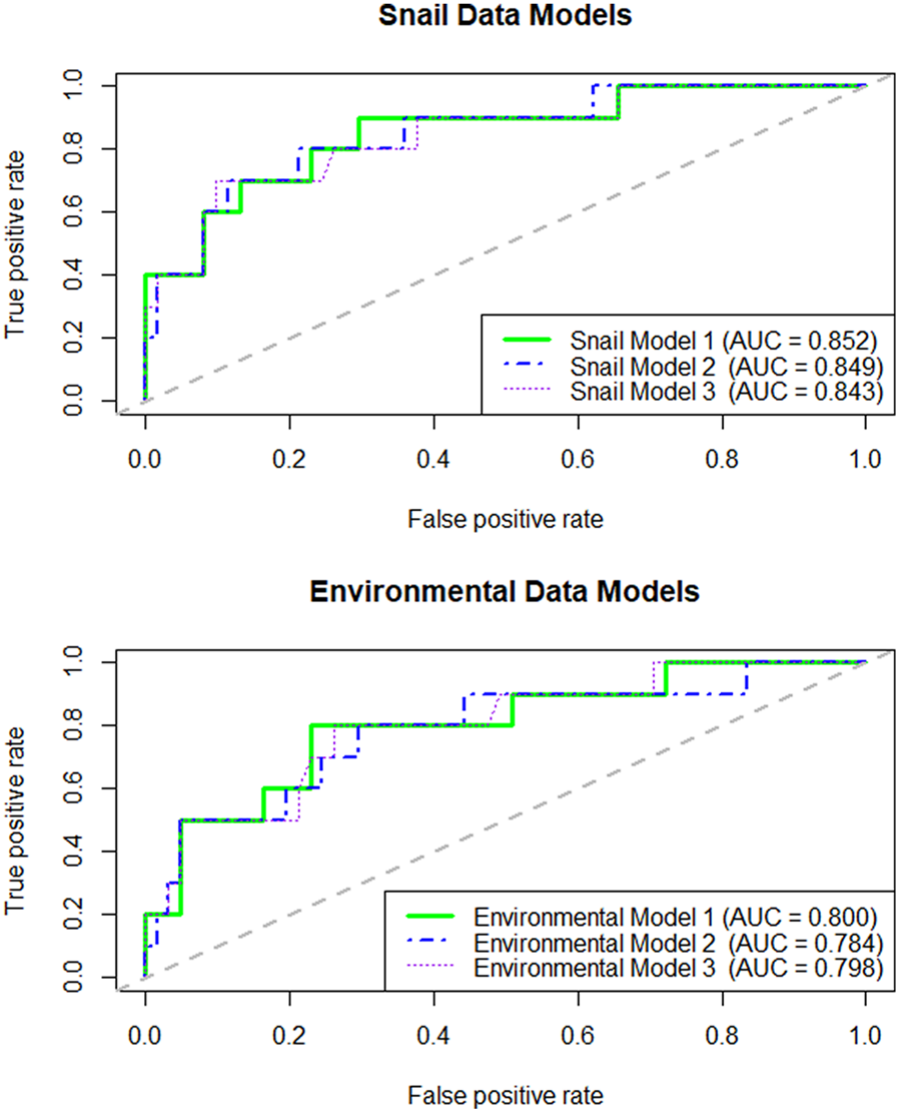
Receiver Operating Characteristics (ROC) Area Under the Curve (AUC) for snail and environmental models

The snail data models were outperformed by the open-source environmental data models using each model’s kappa and accuracy metrics (Table 3, Fig. 3). The accuracy of the best snail model was the same as the NIR of 0.86, which indicates what the accuracy would be expected to be if the majority class were predicted every time. Despite being outperformed in all other metrics, the ROC AUC of each snail model was higher than that of the environmental data models. According to the guidelines laid out by Landis & Koch (1977) on how to interpret the kappa statistic, the kappa values in our snail models (0.33–0.37) all suggest a “Fair” predictive capacity (0.21—0.40) [49]. The sensitivity and the PPV were low for the snail models, with a sensitivity of 0.40 and a PPV of between 0.44–0.50 for all snail models.

**Table 3:** Performance metrics for the snail and environmental data models

Performance metrics	Snail survey data models			Open-source environmental data models		
	Model 1	Model 2	Model 3	Model 1	Model 2	Model 3
AUC	0.852	0.849	0.843	0.800	0.784	0.798
Accuracy	0.845	0.859	0.845	0.887	0.887	0.887
Accuracy 95% CI	0.74–0.92	0.76–0.93	0.74–0.92	0.79–0.95	0.79–0.95	0.79–0.95
NIR^a^	0.859	0.859	0.859	0.859	0.859	0.859
P-Value (Accuracy > NIR)	0.706	0.583	0.706	0.316	0.316	0.316
Kappa^b^	0.332	0.365	0.332	0.492	0.492	0.492
Sensitivity	0.400	0.400	0.400	0.500	0.500	0.500
Specificity	0.918	0.934	0.918	0.951	0.951	0.951
Pos Pred Value	0.444	0.500	0.444	0.625	0.625	0.625
Neg Pred Value	0.903	0.905	0.903	0.921	0.921	0.921

^a^No Information Rate

^b^Due to the high degree of imbalance between the outcome classes across the study period, the Cohen’s kappa statistic is a useful metric for our models, as it helps to correct bias that results when rewarding the prediction of the majority class. The benchmark values outlined by Landis & Koch (1977) are useful here for determining the relative strength of the predictive models: < 0.00 = Poor; 0.00–0.20 = Slight; 0.21–0.40 = Fair; 0.41–0.60 = Moderate; 0.61–0.81 = Substantial; 0.81–1.0 = Almost Perfect

By comparison, the performance metrics of the environmental models indicated strong predictive performance. The accuracy of all three environmental data models was 0.89 (slightly higher than the NIR of 0.86), while the kappa statistic was 0.49, indicating the predictive capacity of the environmental models was “Moderate” using the Landis & Koch benchmarks [49]. The ROC AUC for the environmental models ranged from 0.78–0.80. Although the sensitivity and PPV for the environmental predictor models was still relatively low (sensitivity: 0.50; PPV: 0.63), the specificity (0.95) and NPV (0.92) for all three models were very high.

### Variable importance

**Fig. 4:**
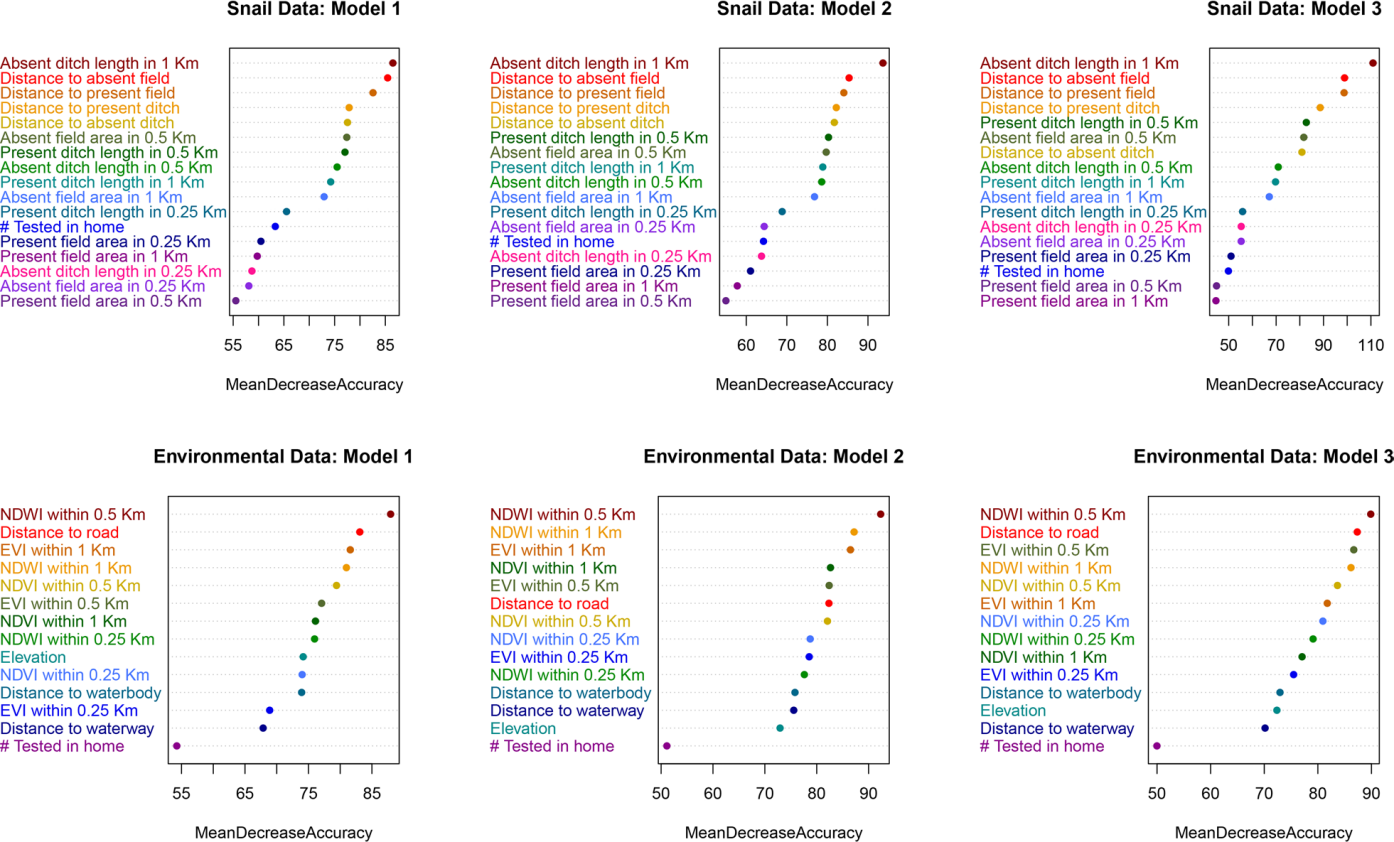
Variable importance plots for the snail and environmental data models. For each of the three models generated with the snail data and the environmental data, variable importance was determined using Mean Decrease in Accuracy (MDA). Each variable is assigned one color across all three models such that color can be used to highlight major shifts in variable importance ranks between models

The variable importance assessment highlighted several key predictors of household *S. japonicum* infection in our study area in 2016. From the environmental data models, mean NDWI within 0.5 km of the home was the best performing predictor, resulting in a three-model summary score of 30 (Table 4 and Fig. 4). Distance to the nearest road and the mean NDWI within 1 km of the home were the next most important predictors, each with a summary score of 23. None of the variables that used a 0.25 km buffer around the home was ranked in the top 50% of predictors, nor was elevation, the distance to waterbodies or waterways, or the number of people tested per household. In the snail survey data models, the total length of all absent ditches (i.e., ditches where no snails were found) within 1 km of the home was the top predictor for all three models, followed by the distance to the nearest absent field and the distance to the nearest present field. Like what was found with the environmental data models, none of the variables that used the smallest household buffer size (0.25 km) were ranked among the top 50% of predictors in the three-model summary score.

**Table 4:**
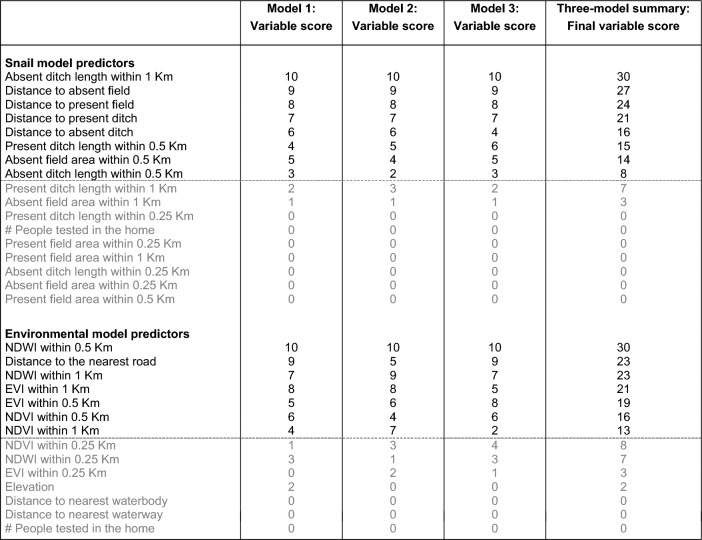
Summary of variable importance rankings for the snail and environmental data models

After dividing the snail habitat data (top), and the environmental data (bottom) 75:25 for training and validation, three balanced training datasets were obtained for each by oversampling the minority outcome class. These balancing repetitions were used to assess the stability of model performance metrics and variable importance rankings that resulted from using an oversampling approach to create a balanced training dataset. After tuning each model using ten-fold cross-validation, the final models were run on the reserved testing data to generate model performance metrics and variable importance summaries (indicated by the Mean Decrease in Accuracy (MDA)). The ten predictors with the highest MDA in each model were given a score of 10 – 1 (10 being the score of the predictor with the highest MDA). Variable scores were then summed across the three models to create a three-model summary score of 30 – 0, 30 being the highest score possible (ranked first in all three models), while a score of 0 indicates that the variable was not ranked in the top ten in any of the three models. In this table, the top ~ 50% of predictors (determined by the three-model summary score) are shown above the dotted line in black, while those that were in the bottom 50% are below the dotted line and shown in gray

### Logistic regressions and predictions

In our simple logistic regression analyses, we found that the total distance to the nearest road was the only predictor from the environmental dataset that was ranked among the top 50% of predictors that was also significantly (p-value < 0.05) associated with household *S. japonicum* infection status (Table 5). For each 1 km increase in the distance between the home and the nearest road, the log odds of household infection increased by 1.30 (standard error (SE) = 0.60, p-value = 0.03). NDWI and EVI within 0.5 km and 1 km of the home were positively associated with household infection status, whereas NDVI was negatively associated with infection status, though none of these associations were statistically significant using p-value < 0.05. Although neither distance to the nearest waterway nor elevation were ranked among the top 50% of the environmental predictors, both were strongly negatively associated with household infection status (p-value < 0.01).

**Table 5:**
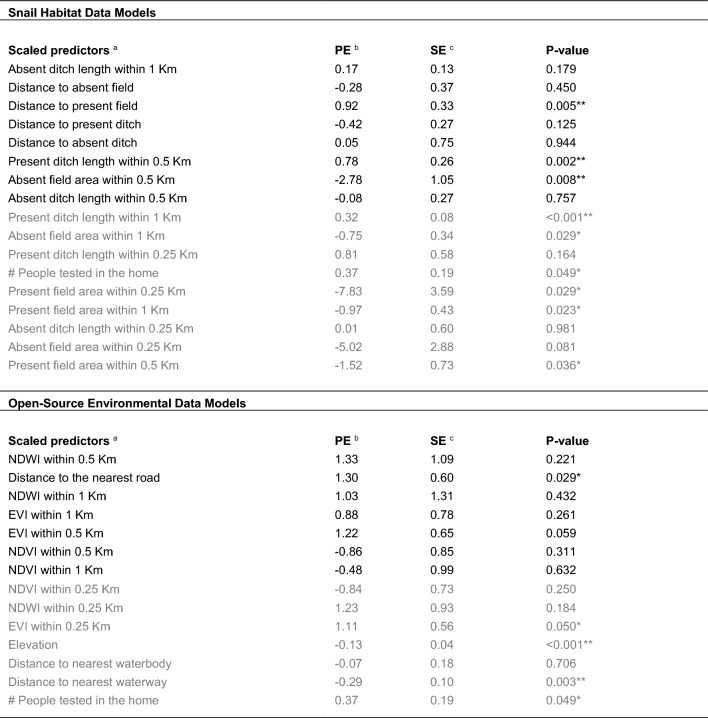
Simple logistic regression results

^a^For both the snail habitat data (top), and the environmental predictors data (bottom), simple logistic regression models were run to determine the direction of association with household *S. japonicum* infection status. Each predictor was scaled to make a one-unit change represent meaningful incremental changes. The units used for each snail variable are as follows: for the distance to the nearest present ditch, absent ditch, present field and absent field, the unit of change was 1 km; for the total present ditch length and total absent ditch length within 0.25 km, 0.5 km and 1 km of the home, the unit of change was 1 km; for the area of present fields and area of absent fields within 0.25 km, 0.5 km and 1 km of the home, the unit of change was 0.1 km^2^; the unit of change was 1 person. The units used for each environmental variable are as follows: for NDWI, NDVI and EVI, the unit of change was 0.1 (index range of -1 to + 1); for the distance to the nearest road, waterway or waterbody, the unit of change was 1 km; for elevation, the unit of change was 10 m; for the number of people tested in the home, the unit of change was 1 person

^b^Point estimate

^c^Standard error

* p-value ≤ 0.05

**p-value ≤ 0.01

In models using snail survey data, ditches were associated with an increased risk of infection while fields were associated with a lower risk of infection. For every 1 km increase in the length of ditches where snails were found within a 1 km radius of the home, the log odds of household infection increased 0.78 (SE = 0.26, p = 0.002). In contrast, infections were more likely in households further from fields: for each 1 km increase in the distance between the home and a field where snails were present, the log odds of household infection increased 0.92 (SE = 0.33, p = 0.005). Likewise, for each 0.1 km^2^ increase in fields where no snails were found within 0.5 km of the home, the log odds of household infection decreased 2.78 (SE = 1.05, p = 0.008). See Table 5 for details on the simple logistic regression results.

**Fig. 5:**
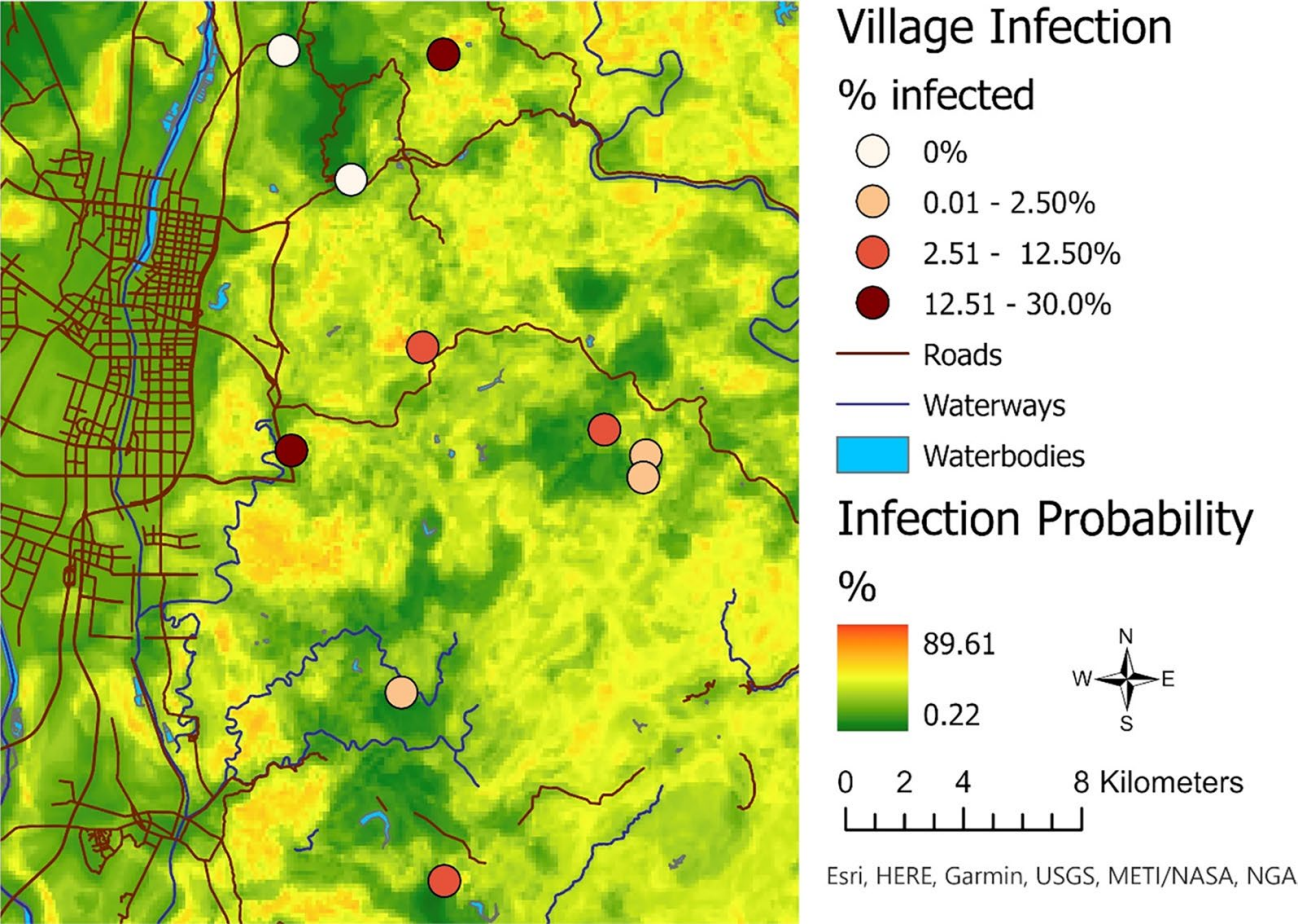
Prediction map showing the probability of *S. japonicum* infection using the top-performing environmental data model. The final top performing model was defined as the one with the highest kappa, accuracy, and receiver operating characteristic (ROC) area under the curve (AUC), respectively. Model performance metrics (Cohen’s kappa and accuracy) highlighted that the open-source environmental data models outperformed the snail data models. The top performing environmental data model was used to create a prediction surface of the probability of *S. japonicum* infection across the entire study area

Given that the kappa and accuracy of the three final environmental data models was higher than the kappa and accuracy of the snail data models, the environmental model with the highest ROC AUC (Model 1; see Table 3) was used as our final prediction model. Using the final model, we generated a prediction surface for the entire study area to illustrate the predicted probability of infection across different landscapes within the study area (Fig. 5). The predicted probability of infection for the study area ranged from 0.2% to 89.6%.

## Discussion

In this study, we set out to gain a better understanding of the strengths and limitations of on-the-ground-surveillance as compared to remote sensing and open-source environmental data for identifying pockets of schistosomiasis in a region approaching elimination. We found that the open-source environmental data models outperformed the snail data models in predicting household *S. japonicum* infection status in rural farming communities in Sichuan, China. Across our models, the sensitivity, specificity, NPV, PPV, kappa and accuracy of the environmental data models was higher than the snail data models. This has important implications. Whereas snail surveys are labor-intensive and time-consuming pursuits, the data from the environmental predictors models are readily available and free to download. Thus, for the purposes of estimating local infection risk in areas approaching elimination, the ultimate payoff of investing resources into snail surveys may be lower than what could be achieved by limiting field activities to human and animal infection surveys and focusing on environmental conditions that can be sufficiently characterized using open-source environmental data.

As more locations approach elimination goals, intensive prevention and control programs and their schistosomiasis-dedicated teams are likely to be phased out in favor of targeted surveillance and response methods. It is therefore becoming increasingly important to explore a range of lower-input alternatives to snail surveys for monitoring schistosomiasis risk in the years to come. In this study, the high specificity (0.95) and NPV (0.92) of our environmental models) suggests that open-source environmental data serves as an effective alternative to large-scale snail surveys for ruling in the possibility of schistosomiasis infection at fine spatial scales in areas on the verge of elimination. This is useful in the context of resource-limited control programs, in that it can serve as a first step in identifying areas where infections are likely to be present (and, conversely, ruling out areas where infections are unlikely to be found). This can enable the efficient direction of resources such as infection screening, preventative prophylaxis and improved sanitation to areas that are predicted to have high infection probability.

To validate our findings and adapt these methods for use in other settings, a few key actions are recommended. First, investigations of the suitability of different environmental measures for predicting human infection across a range of settings are needed, as snail habitat preferences, suitability and transmission risks may vary substantially from ecosystem to ecosystem [30, 53–55]. While our findings demonstrate the potential utility of using open-source environmental data in lieu of snail habitat survey data, our analysis is focused on ten villages from two endemic counties in China in 2016. It remains to be seen if open-source environmental data performs similarly well in other environments and ecosystems. It is important to replicate this analysis in other endemic regions and for other species of schistosomes to determine how well open-source environmental data predicts household infection across distinct environments. Second, to determine whether open-source environmental predictors and their variable importance rankings are stable over time, it would be beneficial to re-evaluate the predictive capacity of these open-source environmental predictors within similar ecosystems at multiple points in time. Finally, because these analyses require access to environmental data and local cadres of trained GIS professionals proficient in developing and analyzing geospatial data, we recommend that regions approaching schistosomiasis elimination targets invest in building their GIS workforce and toolsets to enable locally tailored GIS-based solutions. Although many of the data sources used in this analysis are available at high resolutions across the globe (NASA’s MODIS imagery library, USGS Landsat imagery, JAXA ALOS World 3D-30 m), OSM data is less consistent in availability and coverage. For example, although shapefiles containing processed OSM data are now available for free from Geofabrik for most countries worldwide, a 2017 study estimated that the worldwide completeness of OSM data was 83%, with approximately 40% of countries having fully mapped street networks [56]. Thus, building on local capacities to leverage open-source data, evaluate local coverage and completeness and fill data gaps where they exist is a recommended next step for countries like China that are nearing schistosomiasis control and elimination targets.

In our secondary analyses, we compared several environmental features on their capacity to predict household infection status in 2016 to provide researchers and control programs with insights on the relative importance of a range of local environmental features at varying spatial scales. In this study, homes that were further from a road were significantly more likely to have one or more *S. japonicum* infection. This finding is consistent with the results of other studies, which have suggested that schistosomiasis infection risk is higher in areas that are further from a city [57, 58], a phenomenon potentially related to lower access to healthcare in more remote locations, as has been suggested elsewhere [59]. Our results also highlighted that residents in homes situated in areas with more surface water nearby have a greater risk of schistosomiasis infection – a phenomenon that could be due to increased opportunities for human exposure to schistosomes through water contact, as has been previously found in China, Brazil and several countries in Sub-Saharan Africa [17, 27, 58, 60–64]. In a similar vein, we found that homes that were closer to waterways, as well as those at lower elevations were significantly more likely to have *S. japonicum* infection than those that were nearer to waterways or situated at higher elevations. The association between low elevation and household infection could potentially be linked to water accumulation at lower elevations, or a greater risk of encountering snails at lower elevations, as has been found in China [25, 33, 65], as well as in Côte D’Ivoire, Nigeria, Kenya, Tanzania and Uganda [66–69]. Taken together, the highly ranked predictors featured in our RF and regression models are consistent with what is known about the important role of water in the schistosomiasis transmission cycle, as well as measures of connectivity and remoteness, and highlight the utility of using measures of surface water accumulation and proximity to major road networks as a simple means of schistosomiasis risk characterization and surveillance.

While the models using snail survey data did not perform as well as the open-source environmental data models, we identified a few key predictors that shed light on the relationship between snail habitat and human infections. First, proximity to and the total length of ditches in the area surrounding the home (0.5 – 1 km radius) were consistently among the top predictors of household *S. japonicum* infection and generally more ditches were associated with greater infection risk. For example, our simple logistic regression models suggest that homes that were closer to and those with a greater density of ditches where snails were present were more likely to have one or more residents with *S. japonicum* infection. This aligns with our expectations, as an increase in the area of snail habitats would be expected to correspond with an increasing number of opportunities to encounter infected snails and become infected, as has been found in other contexts [20]. Second, we found surprising evidence that fields may be protective against *S. japonicum* infection – greater density of fields near the home where snails were present or absent, and proximity to fields where snails were present were all associated with decreased household *S. japonicum* infection risk. While determining why this might be the case was beyond the scope of this study, we hypothesize that it is related to a lower overall density of snails across fields, as compared to ditches where snails are likely more compactly situated. This aligns with the findings of a 2014 study from Brazil, where smaller areas of water accumulation were found to have greater snail concentrations, and were subsequently associated with higher disease prevalence [70].

We assessed which spatial scales were most relevant to household *S. japonicum* infection risk by applying three different buffer sizes (0.25 km, 0.5 km, 1 km) around the home to summarize each of our four main snail habitat predictor categories (present fields, absent fields, present ditches, present ditches), and our three environmental indexes measuring surface water and vegetation (NDWI, NDVI, EVI). For all models, only those predictors that used a 0.5 or 1 km buffer were among the top 50% of predictors. Thus, the strongest predictors of our high-resolution outcome (household-level infection) were characteristics of the neighborhood, rather than the area immediately surrounding the home, a finding that is consistent with other studies from the region, which have highlighted the importance of aggregated or village-scaled measures of *S.japonicum* risk [71, 72]. In light of the recent push to incorporate precision mapping into schistosomiasis surveillance and control programs [73], this is an important consideration. Overall, this highlights the important role that spatial scales can play when assessing predictors of environmentally-mediated diseases like schistosomiasis. As a result, we suggest that future studies and control programs consider a range of potential scales of influence when evaluating environmental risk factors, rather than focusing solely on immediate surroundings.

As the aim of our primary analysis was in essence, proof-of-concept, we believe that our main finding—that open-source, remotely sensed data can serve as a substitute for time and labor-intensive snail survey data as a means of identifying high-risk locations—has the potential to hold global significance and therefore sets a precedent for further investigations to determine the extent of its generalizability. Nevertheless, the relatively small geographic area (~ 700 km^2^) and the cross-sectional nature of the study restrict our ability to draw conclusions about other locations or points in time without further corroboration. Additionally, our small study area also resulted in the exclusion of potentially important environmental predictors (e.g., temperature and precipitation) [16–18, 74–76], as weather would not be expected to vary substantially across 25 km (the maximum distance between any two households in this study). Another limitation of this study was that we had a relatively small sample size (N = 283 households), given the number of predictors included in each model (N = 17 in the snail data models, and N = 14 in the open-source environmental data models). While RF models are well-recognized for being robust to small sample sizes and large predictor sets [77], smaller samples result in reduced power to detect rare events and an increased risk that the sample is unrepresentative of the underlying population. We compensated for this, in part, by running multiple models and summarizing broad-scale trends in performance and variable rankings that held across multiple iterations of model building.

Class imbalance in our outcome variable was another limitation of this study, as misclassification rates tend to increase when using RF models to predict outcomes that do not have roughly equal numbers of observations within each category [78]. Overall, 39/283 (13.8%) households had one or more cases of schistosomiasis. To account for the high degree of class imbalance in our outcome, we oversampled the minority class in the training datasets. However, for the reserved validation dataset, the class imbalance remained, resulting in inflated accuracy measures. As such, we recommend that readers prioritize the kappa statistic over the accuracy measure when considering the performance of our models, as this was developed to help correct for bias due to class imbalance [49].

Another limitation in this analysis is that the variable importance measures were likely impacted by the high degree of correlation between some of our predictors (e.g., two different measures of vegetation, or the three different spatial scales used to develop predictors), as the variable importance rankings that are used in RF models become less reliable when predictors are highly correlated with one another [79]. As such, the relative rankings of predictors should be interpreted with caution, instead looking at broad-scale trends in predictor rankings (e.g., ranked in the top 50% of predictors, versus the bottom 50% of predictors). Finally, it is worth noting that the analysis presented in this paper makes use of two datasets that are inherently incomplete. Regarding the snail survey data, despite the use of standard protocols [32], it’s inevitable that not every snail is going to be detected in each field, ditch or other environmental feature, as snail surveys provide only a snapshot of highly dynamic snail populations. The OSM data used in this analysis also has notably gaps, particularly for road networks, for which China is one of the lowest ranked countries for OSM road network completeness, with less than a third of all roads mapped as of 2016 [56]. Nevertheless, the OSM data for China still has been demonstrated to have good coverage and accuracy for major environmental features [36], making them useful for applications such as these where our interest was in developing simple proxy measures for things like the relative remoteness of the home, or the likelihood of water contact. As such, both our snail survey dataset and our open-source environmental dataset are likely to represent typical data for the area, making this analysis an assessment of the predictive capacity of two real-world datasets, which have each been shown to perform reasonably well in predicting household schistosomiasis risk, despite their limitations.

## Conclusion

In this study, we compared the use of labor-intensive snail survey data with that of open-source environmental data for developing prediction models aimed at predicting household infection status among rural farming communities in China. Overall, we found that freely available environmental data can be used to predict household infection status among rural farming communities in Sichuan Province, China, with high accuracy. Furthermore, the open-source environmental data ultimately outperformed the snail habitat data, suggesting that, prior to conducting comprehensive snail surveys, the overarching goal of the surveys ought to be considered to determine whether less resource-intensive methods might be suitable. Not only has this analysis helped to improve our understanding of where and when transmission is most likely to be occurring in the study area, but it has also highlighted specific aspects of the local environment that were associated with household infection—for example, homes that were furthest from roads, or those surrounded by more surface water—which can become the target of future surveillance and control efforts. Replication of this study in other contexts can help assess the generalizability of our findings and allow the development of context-specific models. As the global schistosomiasis community searches for new approaches to identify residual transmission hotspots and maximize the impact of schistosomiasis surveillance efforts, this study shows the potential of using open-source environmental data to generate high-precision schistosomiasis risk maps.

## Supplementary Information

**Additional file 1.** The process and ArcGIS Pro tools used to form each of our variables, as well as the rationale for their formulation and inclusion in this analysis are described in detail in Additional File 1.

## Data Availability

The datasets used in this analysis have not been made publicly available because they correspond with household coordinates and private health information of study participants; therefore data will not be made publicly available for confidentiality reasons.

## Abbreviations

*GPS*: Global Positioning System
*GIS*: Geographic Information System
*OSM*: OpenStreetMap
*JAXA*: Japan Aerospace Exploration Agency
*EORC*: Earth Observation Research Center
*ALOS*: Advanced Land Observing Satellite
*USGS*: United States Geological Survey
*EROS*: Earth Resources Observation and Science Center
*NASA*: National Aeronautics and Space Administration
*MODIS*: Moderate Resolution Imaging Spectroradiometer
*NDWI*: Normalized Difference Water Index
*NDVI*: Normalized Difference Vegetation Index
*EVI*: Enhanced Vegetation Index
*RF*: Random Forests
*ROC*: Receiver Operator Curve
*AUC*: Area Under the Curve
*PPV*: Positive Predictive Value
*NPV*: Negative Predictive Value
*NIR*: No Information Rate
*MDA*: Mean Decrease in Accuracy
*SD*: Standard Deviation
*SE*: Standard Error
