# Supplementary material for: Open-source environmental data as an alternative to snail surveys to assess schistosomiasis risk in areas approaching elimination
Source: Int J Health Geogr. 2023 Jun 2;22:12. doi: 10.1186/s12942-023-00331-w (PMC10236814; doi:10.1186/s12942-023-00331-w)
Supplement: Supplementary file 1 — Additional file 1. The process and ArcGIS Pro tools used to form each of our variables, as well as the rationale for their formulation and inclusion in this analysis are described in detail in Additional File 1. [file 12942_2023_331_MOESM1_ESM.docx]

**Additional File 1. Detailed description of variable rationale and definitions, the data generation process and ArcGIS Pro tools used to form the geospatial datasets and prediction map.**

*Outcome variable*

*S. japonicum* infection survey results from the ten study villages were aggregated to the household level and spatially joined to the geographic location of the home. To avoid issues with multicollinearity resulting from residents of the same household having the same values for all environmental predictors used in this analysis, the outcome was a binary measure of household infection status, with “0” indicating no infections detected among participating household members, and “1” indicating that one or more household member tested *S. japonicum* positive. Each household was represented in the geospatial dataset as a point feature.

*Predictor variables: snail survey dataset*

The geocoded snail habitat data collected during national surveys in 2016 was first divided into two categories: present snail habitat sites, and absent snail habitat sites. Present snail habitat sites were those sites where one or more snails were identified during the survey period, while absent snail habitat sites were those where snails were not found during the 2016 survey. For both present and absent sites, the environment type was recorded as either a polygon or line feature in the dataset. Polygons most frequently represented rice paddy fields in the area, though they occasionally represented other habitat sites such as a small pond, a dry field or a beach. Meanwhile, line features most commonly represented dirt or concrete irrigation ditches used for flooding rice paddies, though it could also correspond with streams or other narrow waterways. For the purposes of this analysis, all polygons are referred to as “fields”, while all lines are referred to as “ditches”. This classification system resulted in a total of four snail habitat categories: present ditches, present fields, absent ditches, and absent fields.

Using ArcGIS Pro software (1), three different buffer sizes (0.25, 0.5 and 1.0 kilometer (km) radius length) were generated and applied to each household location using the “Buffer” analysis tool. These buffer radius lengths were defined such that the largest buffer (1 km) generally spanned the entire village area for a centrally located household, whereas the smallest buffer (0.25 km) spanned the immediate surroundings of a given household. The “Summarize Within” analysis tool was used to calculate the total length (km) of present ditches and absent ditches that fell inside each household buffer area. This step was repeated for the present fields and absent fields, calculating the total area of fields (km^2^) that were encapsulated by each household buffer. The “Near” analysis tool was then used to calculate the geodesic distance (in meters) between each household point and the nearest present ditch, absent ditch, present field, and absent field. The “Join Field” data management tool was used to join all of the newly created variables summarizing the length and area of ditches and fields into a single table, which was then exported to the project’s geodatabase for use in R. Including separate measures for the distance to the nearest ditch and nearest field, as well as the total length and area of ditches and fields within a given buffer area helped us determine whether these features varied in their relative predictive capacity or in their respective spatial scales (i.e. buffer sizes) of influence.

*Predictor variables: open-source environmental dataset*

Open-source environmental and remotely sensed data were compiled to create a geospatial dataset containing a range of hypothesized environmental (built and natural) predictors of household *S. japonicum* infection. Potential environmental predictors were selected if they were 1) previously identified or hypothesized in the literature to serve as predictors of schistosomiasis infection or snail habitat sites; and 2) made publicly available at a 250-meter resolution or finer for the entire study area. Most of the predictors represented natural features of the environment (e.g., waterbodies, elevation, vegetation indices, etc.). Human-made environmental features like roads were also included, as the relative remoteness or connectedness of a given household was hypothesized to be a factor associated with schistosomiasis infection status. Roads and waterways from the OpenStreetMap (OSM) project were included as line features, while waterbodies were water features coded as polygons. The “Near” Analysis tool was then used to calculate the geodesic distance (km) between each household and the nearest road, waterway, or waterbody.

Prior studies have suggested that elevation is negatively associated with the presence of *O. hupensis* snails (2, 3). We used 30-meter resolution elevation data from JAXA EORC’s ALOS satellite to extract the elevation (m) value that corresponded with each household point location.

The presence of either water or vegetation can provide opportunities for water contact and have the potential to impact human infection risk. In this study, we use the Normalized Difference in Water Index (NDWI) (4) to estimate water content across the study area, and the Normalized Difference in Vegetation Index (NDVI) (5) and the Enhanced Vegetation Index (EVI) (6) to describe vegetation health and density in the study area. The NDWI identifies water features and distinguishes them from soil and vegetation surfaces (4). The NDVI index is chlorophyll-sensitive and provides a measure of crop and vegetation health, while the EVI is more sensitive to canopy variations and performs particularly well in high biomass regions (7). As such, the two vegetation measures complement each other and are frequently used jointly in vegetation studies (7). Whereas NDWI and NDVI were calculated using data from the Landsat-8, Collection 1, pre-processed EVI data at 250-meter resolution was downloaded directly from NASA’s MODIS data library (8).

There was a total of 12 Landsat-8 satellite images collected between January and July of 2016, all of which were examined and processed to remove all medium to high confidence clouds, cloud shadows or other sources of terrain occlusion. To do this, the QA band files made available by USGS for each of the Landsat-8 observations (9) were used in ArcGIS Pro with the “Remap” raster function, to recode all grid cells corresponding with terrain occlusion as “No Data”, while all clear or low confidence cloud cells were set to equal 1. The “Clip” Raster function was then used to remove all cells obscured by cloud cover from each corresponding Landsat-8 image. Overall, cloud cover was high between January 2016 – July 2016 over the study area, ranging from 9.97 – 100%, with an average cloud coverage of 66.13% across the 12 satellite observations made within that period. To assess the extent to which the removal of cloud cover and terrain occlusion would result in missing data for each household at each time point, the “Raster to Points” tool was used to convert the cloud-corrected satellite data to a grid of points, and the “Extract Multi Values to Points” geoprocessing tool was used to extract the data corresponding to each household’s point location. A total of 3 of the 12 Landsat-8 collections had <30% cloud coverage and had cloud-corrected data available for between 98.5 – 100% of households. The remaining 9 collections had cloud cover ranging from 33 – 100%, which resulted in data for between 0 – 65% of households. As such, we restricted our use of the Landsat data to the 3 collections with < 30% cloud coverage (collected on January 23^rd^, February 8^th^, and April 28^th^ of 2016).

Using ArcGIS Pro’s “Raster Calculator” Image Analyst tool, the NDWI on January 23^rd^, February 8^th^ and April 28^th^ were each calculated from the cloud-corrected Green and Near Infrared (NIFR) Landsat Surface Reflectance bands (bands 3 and 5 in Landsat-8, respectively), using the following formula developed by McFeeters (1996) (4):

$$NDWI=\frac{\left( Green-NIFR \right)}{(Green+NIFR)}$$

NDVI on January 23^rd^, February 8^th^ and April 28^th^ was calculated from the cloud-corrected Red and NIFR Landsat Surface Reflectance bands (bands 4 and 5 in Landsat-8, respectively), using the following formula (5):

$$NDVI=\frac{\left( NIFR-Red \right)}{(NIFR+Red)}$$

The “Mosaic to New Raster” tool was then used to calculate an estimate of the average NDWI and NDVI across the three time points with sufficient data coverage.

EVI is calculated based on Blue, Red and NIFR Reflectance bands, as well as a soil adjustment factor (L), and two coefficients (C_1_ and C_2_) used to correct for aerosol scattering, as shown in the following formula (5, 7):

$$EVI= (1+L)*\frac{\left( NIR - Red \right)}{(NIFR +(C1*Red) -(C2 * Blue) + L)}$$

All 12 of the pre-processed, cloud-corrected EVI measures at 16-day intervals for the period between January – July 2016 were downloaded from NASA’s MODIS Terra Satellite Imagery database (8), and were joined into a single, average EVI measure using the Mosaic to New Raster” tool in ArcGIS Pro.

Each of the final NDWI, NDVI and EVI measures were converted to a grid of points using the “Raster to Multi Points” tool. As was done for the snail data, three different buffers sizes were generated around each household point (0.25, 0.5 and 1.0 km radius), and the “Summarize Within” analysis tool was then used to calculate the average NDWI, NDVI and EVI in the 0.25, 0.5 and 1 km area surround each household. Not only did this make our measures of NDVI, NDWI and EVI representative of the average conditions surrounding the home, but this also helped to facilitate the comparison between our 30-m resolution measures of NDWI and NDVI and the 250-m resolution measure of EVI.

Sensitivity analyses were also conducted to compare the predictive performance of models that incorporated different temporal scales for formulating NDWI, NDVI and EVI. In our first model, we used all available satellite observations with cloud coverage of <30% from 2016 to estimate NDWI, NDVI and EVI. In subsequent models, we restricted our calculations of NDVI, NDWI and EVI to using only satellite observations from later months (e.g. April – July), or satellite observations where 100% of households had cloud-corrected data available. As performance was similar across all models, our final model used all available satellite observations with cloud coverage of <30% from 2016 to estimate NDWI, NDVI and EVI.

*Prediction mapping*

Using the top performing RF model, a map of the predicted probability of *S. japonicum* infection across the entire study area was generated. Within ArcGIS Pro, the “Raster to Point” tool was used to generate a grid of points covering the entire study area surface. The grid dataset was then exported to R using the R-ArcGIS Bridge to calculate the predicted probability of infection at each point across the study area. These predicted probabilities were added to the grid dataset and exported back to ArcGIS Pro for mapping. Finally, the “Point to Raster” tool was used to transform the predicted probabilities into a raster surface, using the “Mean” method for the cell assignment type.

**References**

1. Esri Inc. ArcGIS Pro 2.8.3 2021 [Available from: <https://www.esri.com/en-us/arcgis/products/arcgis-pro/overview>.

2. Qiu J, Li R, Xu X, Yu C, Xia X, Hong X, et al. Identifying determinants of Oncomelania hupensis habitats and assessing the effects of environmental control strategies in the plain regions with the waterway network of China at the microscale. International journal of environmental research and public health. 2014;11(6):6571-85.

3. Shan X, Liu S, Liu J, Zhu H, Xiao Y, Chen Y. Geographical survey of the intermediate host of Schistosoma japonicum: Toward precise management of Oncomelania hupensis. PLOS Neglected Tropical Diseases. 2020;14(10):e0008674.

4. McFeeters SK. The use of the Normalized Difference Water Index (NDWI) in the delineation of open water features. International Journal of Remote Sensing. 1996;17(7):1425-32.

5. Rouse JW, Haas RH, Schell JA, Deering DW, editors. Monitoring vegetation systems in the great plains with ERTS. 3rd Earth Resource Technology Satellite (ERTS) Symposium Proceedings; 1973.

6. Gao X, Huete AR, Ni W, Miura T. Optical–Biophysical Relationships of Vegetation Spectra without Background Contamination. Remote Sensing of Environment. 2000;74(3):609-20.

7. Huete A, Didan K, Miura T, Rodriguez EP, Gao X, Ferreira LG. Overview of the radiometric and biophysical performance of the MODIS vegetation indices. Remote Sensing of Environment. 2002;83(1):195-213.

8. NASA. MODIS Vegetation Index Products (NDVI and EVI) 2022 [Available from: <https://modis.gsfc.nasa.gov/data/dataprod/mod13.php>.

9. National Aeronautics and Space Administration (NASA). Landsat Overview 2021 [updated December 9th, 2021. Available from: <https://www.nasa.gov/mission_pages/landsat/overview/index.html>.
